# Baseline susceptibility of *Anopheles gambiae* to clothianidin in northern Ghana

**DOI:** 10.1186/s12936-023-04769-y

**Published:** 2024-01-09

**Authors:** Cosmos M. Pambit Zong, Sylvester Coleman, Abdul Rahim Mohammed, Christopher M. Owusu-Asenso, Yaw Akuamoah-Boateng, Isaac K. Sraku, Simon K. Attah, Liwang Cui, Yaw A. Afrane

**Affiliations:** 1 Department of Medical Microbiology, University of Ghana Medical School, University of Ghana, Korle-Bu, Accra, Ghana; 2 Department of Clinical Microbiology, Department of Clinical Microbiology, College of Health Sciences, Kwame Nkrumah University of Science and Technology, Kumasi, Ghana; 3 Department of Internal Medicine, University of South Florida, 3720 Spectrum Blvd, Tampa, FL 33612 USA

**Keywords:** *Anopheles gambiae* s.l., Insecticide resistance, Target-site mutation, Clothianidin, Indoor residual spraying, Ghana

## Abstract

**Background:**

Clothianidin, an insecticide with a novel mode of action, has been deployed in the annual indoor residual spraying programme in northern Ghana since March 2021. To inform pragmatic management strategies and guide future studies, baseline data on local *Anopheles gambiae* sensu lato (s.l.) susceptibility to the clothianidin insecticide were collected in Kpalsogu, a village in the Northern region, Ghana.

**Methods:**

Phenotypic susceptibility of *An. gambiae* mosquitoes to clothianidin was assessed using the World Health Organization (WHO) insecticide resistance monitoring bioassay. The WHO cone bioassays were conducted on mud and cement walls sprayed with Sumishield 50 wettable granules (WG) (with clothianidin active ingredient). Daily mortalities were recorded for up to 7 days to observe for delayed mortalities. Polymerase chain reaction (PCR) technique was used to differentiate the sibling species of the *An. gambiae* complex and also for the detection of knock down resistance genes (*kdr*) and the insensitive acetylcholinesterase mutation (*ace-1*).

**Results:**

The WHO susceptibility bioassay revealed a delayed killing effect of clothianidin. Mosquitoes exposed to the cone bioassays for 5 min died 120 h after exposure. Slightly higher mortalities were observed in mosquitoes exposed to clothianidin-treated cement wall surfaces than mosquitoes exposed to mud wall surfaces. The *kdr* target-site mutation L1014F occurred at very high frequencies (0.89–0.94) across all vector species identified whereas the *ace-1* mutation occurred at moderate levels (0.32–0.44). *Anopheles gambiae* sensu stricto was the most abundant species observed at 63%, whereas *Anopheles arabiensis* was the least observed at 9%.

**Conclusions:**

*Anopheles gambiae* s.l. mosquitoes in northern Ghana were susceptible to clothianidin. They harboured *kdr* mutations at high frequencies. The *ace-1* mutation occurred in moderation. The results of this study confirm that clothianidin is an effective active ingredient and should be utilized in malaria vector control interventions.

## Background

Malaria is endemic in all parts of Ghana, with seasonal variations being more pronounced in the Northern parts of Ghana [1]. In Ghana, malaria cases account for 10.4 million outpatient visits per year, 4.2% of which are children under the age of 5 [2].

Global malaria control and elimination efforts rely heavily on vector control interventions. Indoor residual spraying (IRS) and insecticide-treated nets (ITNs) are the principal malaria vector control tools [3, 4]. These vector-based control measures have been deployed in malaria-endemic communities with high vector densities and high transmission rates in sub-Saharan Africa, resulting in significant declines in malaria burden in sub-Saharan Africa in the past decade [5]. At total of 67% of households in Ghana owned at least one ITNs in 2022. 47% of households in Ghana had full ITN coverage [6]. In Ghana, IRS is currently being implemented in 23 administrative districts, mainly in northern Ghana, where malaria transmission has been very seasonal and high [6]. Over the years, lambda-cyhalothrin (pyrethroid), deltamethrin (pyrethroid) and pirimiphos-methyl (organophosphate) insecticides have been successively utilized in the IRS programmes [7].

Intensive insecticide application and over-reliance on a narrow spectrum of insecticide classes have imposed an intense selection pressure over time, causing malaria vector populations to develop resistance to these insecticides [8, 9], which is hampering vector control efforts. *Anopheles gambiae* sensu lato (s.l.) resistance to organochlorides and pyrethroids, is highest in West Africa and widespread in Ghana [10–13]. Studies in Ghana [10, 14] have reported on target site insecticide resistance mechanisms namely; *kdr*-L1014F, kdr-L1014S, *N1575Y ace-1* (G119S) and metabolic resistance in *Anopheles* mosquitoes from Northern Ghana.

Recent reports of insecticide resistance to pirimiphos-methyl [15, 16], an organophosphate, have compelled the appropriate authorities to act in response to these insecticide resistance concerns. Hence, the Innovative Vector Control Consortium (IVCC), Insecticide Resistance Action Committee (IRAC) and the World Health Organization (WHO) have introduced a new class of insecticide (neonicotinoid) for malaria control programmes [17].

In response to insecticide resistance concerns, the National Malaria Elimination Programme (NMEP) through the US President’s Malaria Initiative has introduced a new insecticide, clothianidin for IRS in northern Ghana. SumiShield™ 50WG (containing clothianidin as the active ingredient) and Fludora Fusion™ WP-SB (active ingredient: clothianidin and deltamethrin), are recommended by the WHO for use in IRS to deliver insecticidal activity in malaria vectors via tarsal contact [18, 19].

In order to efficiently manage IRS and ultimately help the country meet the global malaria goal of eliminating malaria by 2030 [20], it is important to have up-to-date information on local vector susceptibility and resistance trends in order to inform the NMEP to make timely changes of the insecticides. This study, therefore, sought to determine local vector susceptibility to clothianidin in northern Ghana.

## Methods

### Study site

**Fig. 1:**
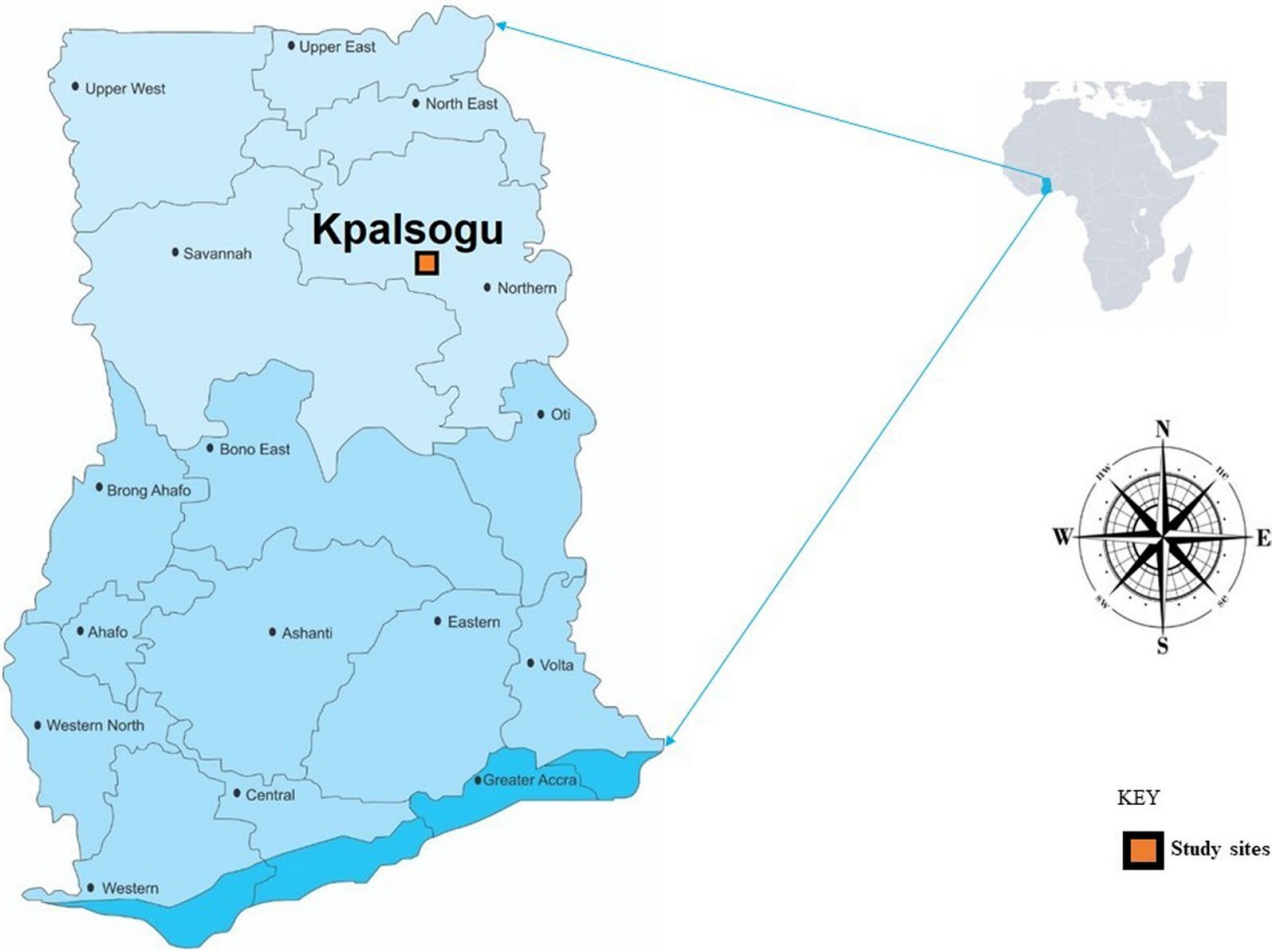
A map of Ghana showing the study site

The study was carried out in Kpalsogu (09° 24ʹ 27″ N 00° 51ʹ 12″ W), a rural community in the Kumbungu District, northern Ghana (Fig. 1) where IRS with SumiShield™ 50WG is implemented.

In this region the annual average temperature ranges from 24 to 34 ºC. The region has a unimodal rainfall pattern, with the rainy season occurring from May through November and the dry season from December to April. There is a large irrigation dam (Botanga irrigation dam) in this area which serves 13 communities [21], with several trenches and furrows irrigating the farms of the inhabitants, creating breeding habitats for mosquitoes.

### Sampling of *Anopheles gambiae* immatures

*Anopheles gambiae* larvae were sampled from identified breeding sites in the community from April 2021 to August 2021. Geographical positioning coordinates and characteristics of the habitats (land use and vegetation) were documented. Samples were subsequently transported to the insectary of the PMI Vectorlink project in Tamale, Ghana, where they were raised into adults. Larvae were fed with ground fish meal (Tropical Fish Food Flakes) and under the typical rearing conditions (26 ± 2 °C; 80% ± 10% relative humidity (RH) with 12 h: 12 h light/dark cycle).

Upon pupation, pupae were collected into plastic cups and placed in mosquito cages for the emergence of adult forms which were fed with a 10% sucrose solution. About 3–5 days old female *Anopheles* to be used in the bioassays were held in WHO bioassay holding tubes for an hour to check for fitness. *Anopheles* with broken wings, legs or unable to fly were deemed unfit and discarded.

### WHO insecticide susceptibility bioassays

One hundred and twenty 3–5 day-old unfed female *Anopheles* were used for the bioassays. Four exposure tubes were lined with filter papers impregnated with 2% clothianidin. Two control tubes were also lined with untreated filter papers. Twenty of the selected mosquitoes were placed in each tube and observed for 60 min. Mosquito knockdown scores were recorded at intervals of 10 min during the 60 min exposure period.

After the exposure period, the mosquitoes were transferred into clean holding tubes, and a wad of cotton soaked with 10% sucrose solution was placed on top of it. The mortality rate was checked after 24 h. Subsequently, each tube was checked for dead or alive mosquitoes and scored using the WHO insecticide monitoring guideline [22]. Mosquitoes that survived after 24 h were monitored for delayed mortality for an extra 168 h (7 days). Following the same procedure, a control experiment was conducted using the insecticide with susceptible Kisumu strain of *An. gambiae.*

### WHO Cone bioassays

The cone bioassay was conducted in four different houses (2 with cement wall surfaces and 2 with mud wall surfaces). These walls were sprayed with SumiShield™ 50WG during the annual Indoor Residual Spraying (IRS) programme conducted in March 2021 [23]. Four replicates consisting of 10 female *Anopheles gambiae* mosquitoes aged 3–5 days each were aspirated and transferred into cones that were fixed on walls of rooms sprayed with SumiShield™ 50WG. The mosquitoes were exposed for 5, 10, 20 or 30 min, transferred to plastic cups (10 per cup), and provided with a 10% sucrose solution. Mortality rates were observed for up to 7 days and scored using the WHO cone bioassay guideline [24].

### Identification of *Anopheles gambiae* complex species

A total of 100 (about 10%) of *An. gambiae *s.l. exposed in the tube bioassays were morphologically identified using the keys by Gillies and Coetzee [25]. Individual mosquito legs were cut and used as Deoxyribunucleic Acid (DNA) templates to undertake PCR for discriminating the sibling species using the criteria described by Santolamazza et al. [26].

### Detection of insecticide resistance markers

Extracted DNA from mosquito legs was used to detect the presence of insecticide resistance markers in the mosquitoes using convectional PCR protocols. The markers included Voltage-gated sodium channels (Vgsc-1014 F and Vgsc-1014 S) for target-site resistance mutations to pyrethroids and DDT in voltage-gated sodium channels and *ace 1*-119 S mutation, a marker of resistance to carbamates and organophosphates. Vgsc-1014 F and Vgsc-1014 S were characterized according to Martinez et al. [27] whereas Ace1-119 S was characterized as described by Weil et al. [28].

### Data analysis

Data were analysed using Microsoft Excel 2016. Frequency distribution was used to describe *An. gambiae* s.l. species abundance. Graphs and tables were drawn using Microsoft Excel 2016. The WHO insecticide resistance monitoring guideline was used to define resistance levels. A test population was deemed susceptible if it had a mortality rate of 98% or higher; possibly resistant if the mortality rate was between 90 and 97% and scored as resistant if the mortality rate was lower than 90%.

Abbot’s formula was used to correct mortalities when mortalities in control setups were greater than 5%. Allele frequencies of *kdr* and *ace-1* mutations were calculated. The Hardy-Weinberg equilibrium equation was used to calculate allele frequencies of resistant genotypes. A p-value less than 0.05 was noted as a significant departure from the Hardy-Weinberg equilibrium.

## Results

### WHO insecticide resistance monitoring bioassay

**Fig. 2:**
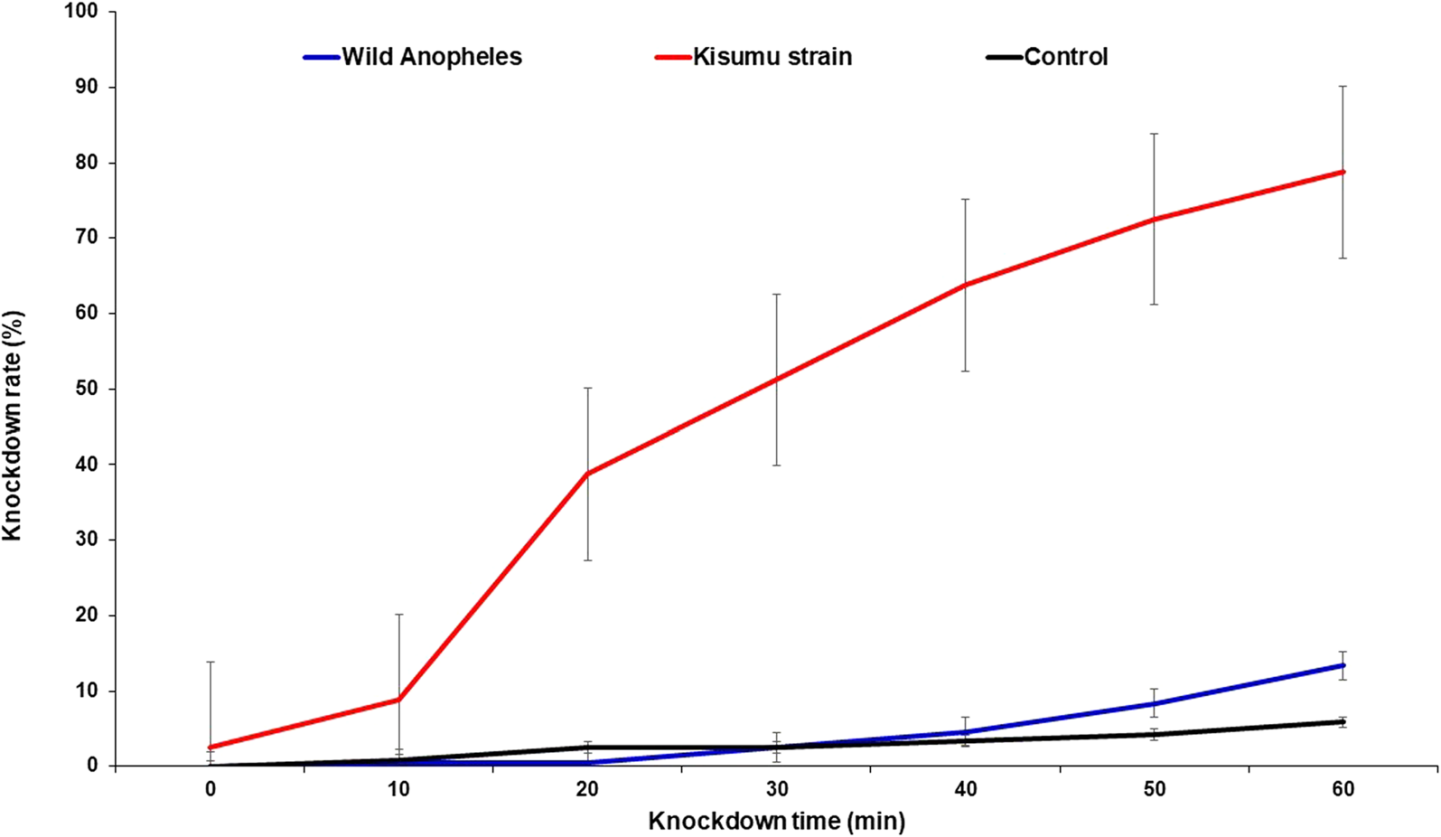
Knockdown rates of wild *Anopheles gambiae* s.l. mosquitoes and Kisumu strain after exposure to clothianidin. Error bars indicate the 95% confidence interval

After 30 min of exposure, less than 5% of the wild *An. gambiae* s.l. were knocked down compared to 51% of the susceptible Kisumu strain at the same time. After 60 min exposure to clothianidin only 13% of the wild population was knocked down compared to 78% of the Kisumu strain (Fig. 2).

**Fig. 3:**
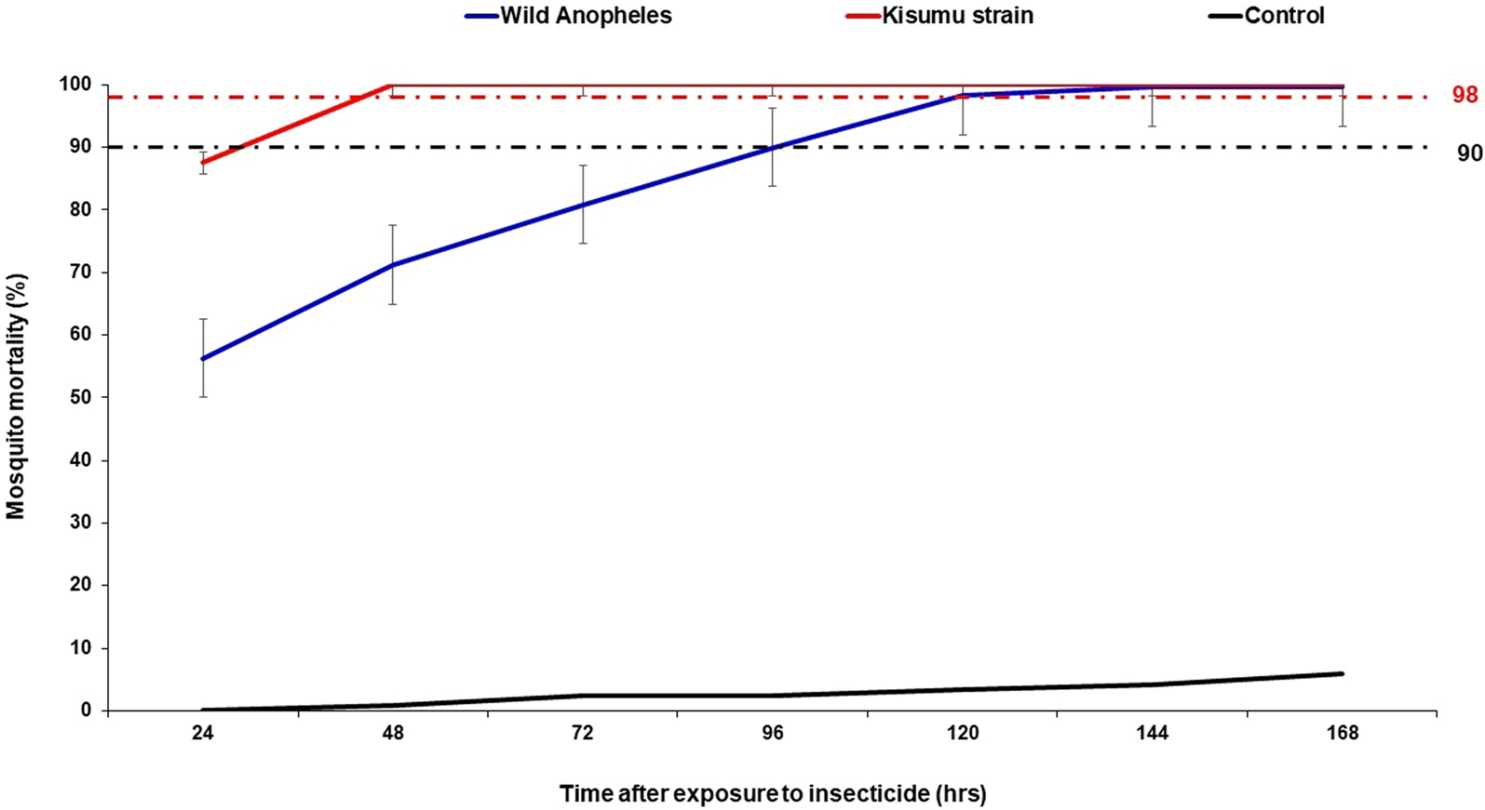
Daily mortality rates of wild *Anopheles gambiae* s.l. and the Kisumu strain following 60-min of exposure to clothianidin. Error bars indicate the 95% confidence interval

Mortality rates observed demonstrated a delayed effect of clothianidin in the wild *Anopheles gambiae* s.l. population. In the wild *An. gambiae*, the mortality rate observed after 24 h was 56% indicative of insecticide resistance. The exposed *An. gambiae* population reached a mortality of 98% after 120 h suggesting susceptibility. In the WHO insecticide resistance monitoring bioassay control test, a 99% mortality rate was observed for the Kisumu strain after 24 h indicating susceptibility (Fig. 3).

### Mortality of mosquitoes exposed to clothianidin treated mud walls

**Fig. 4:**
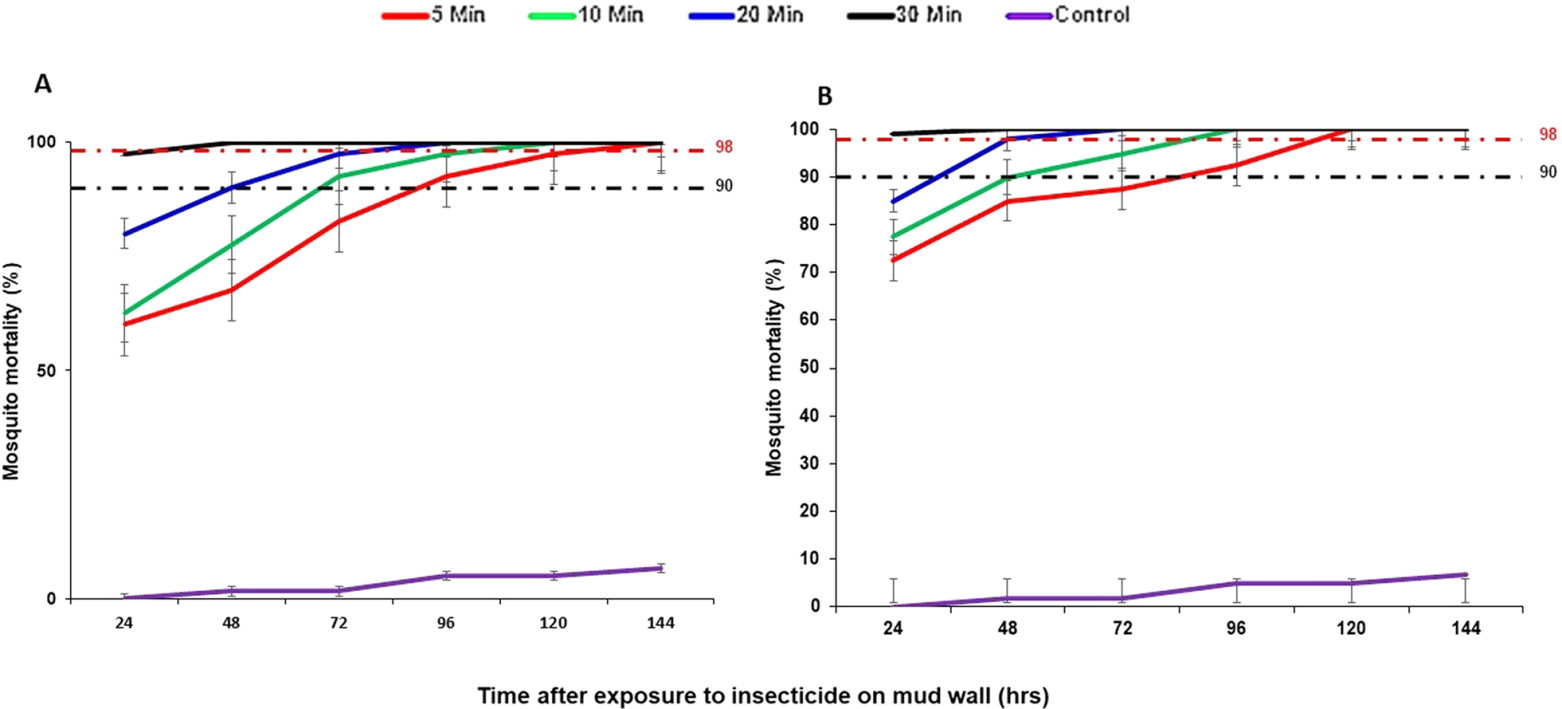
Mortality rates of wild *Anopheles gambiae* s.l. exposed to clothianidin-treated: **A** mud wall and **B** cement wall surfaces. Error bars indicate the 95% confidence interval

For mosquitoes exposed to the clothianidin-treated mud wall surfaces, 5, 10, 20 min of exposure induced 60, 62 and 80% mortality after 24 h, respectively (Fig. 4A). However, the mortality rates of the mosquitoes was 98% after 144, 120 and 96 h, respectively. Finally, 30 min of clothianidin exposure killed all the mosquitoes (100%) after 48 h.

### Mortality of mosquitoes exposed to clothianidin-treated cement walls

Mosquitoes exposed to clothianidin-treated cement wall surfaces for 5, 10 and 20 min had 72, 77 and 85% mortality at 24 h respectively (Fig. 4B). Mosquito mortality rates after these exposure times reached mortality rates of 98% at 120, 96 and 48 h post-exposure, respectively. 100% (100%) mortality was observed at 24 h in mosquitoes exposed to clothianidin for 30 min.

### Species identification and insecticide resistance-mediating mutations of *Anopheles gambiae* complex andcharacterization of insecticide resistance genes

Out of the 100 *An. gambiae* s.l. mosquitoes used for molecular identification of species, 63% were identified as *An. gambiae sensu stricto* (s.s.), 25% were identified as *Anopheles coluzzii* and 9% as *Anopheles arabiensis*. 3% (3%) of the specimen did not amplify by PCR.

The vgsc L1014F mutation occurred at very high frequencies across all vector species identified whereas the *ace-1* mutation occurred at moderate levels (Table 1). The allele frequency of vgsc L1014F was 0.89 in *An. gambiae* s.s. (*χ*^2^ = 0.98, *df* = 60, *P* = 0.32), 0.92 in *An. coluzzii* (*χ*^2^ = 0.19, *df* = 23, *P* = 0.66), and 0.94 in *An. arabiensis* (*χ*^2^ = 0.03, *df* = 6, *P* = 0.86).

**Table 1:** Species composition and distribution of insecticide resistance markers in *Anopheles gambiae* s.l. populations from northern Ghana

Species	Species Composition	*Vgsc*			*Ace-1*		
		Locus 1014		P-value	Locus 119		P-value
		L1014	L1014F		G119	G119S	
*Anopheles gambiae* s.s.	63	0.11	0.89	0.32	0.63	0.37	0.0001
*Anopheles coluzzii*	25	0.08	0.92	0.66	0.68	0.32	0.019
*Anopheles arabiensis*	9	0.06	0.94	0.86	0.56	0.44	0.016

The *ace-1* G119S mutation was also observed in all *An. gambiae* identified with frequency of 0.37 (*χ*^2^ = 20.83, *df* = 61, *P* = 0.0001) in *An. gambiae* s.s. 0.32 (*χ*^2^ = 5.54, *df* = 24, *P* = 0.019) in *An. coluzzii* and 0.44 (*χ*^2^ = 5.71, *df* = 7, *P* = 0.016) in *An. arabiensis*.

## Discussion

Malaria vectors in Ghana have developed resistance to all four classes of insecticides i.e., pyrethroids, organochlorides, carbamates and organophosphates, threatening the sustainability of malaria vector control. The widespread insecticide resistance highlights the need to introduce new insecticide classes with diverse modes of actions to the key malaria vector control interventions. The insecticide clothianidin as an active ingredient of two main formulations was introduced into IRS programme in Ghana.

Here, the susceptibility of *An. gambiae* to this newly introduced insecticide was evaluated as well as the prevalence of insecticide resistance markers. It was observed that, malaria vectors displayed high-level susceptibility to clothianidin, and insecticide resistance mutations were highly prevalent in *An. gambiae* populations in northern Ghana.

The WHO susceptibility test results demonstrated a delayed killing effect of clothianidin on wild *An. gambiae* from northern Ghana. Unlike the neurotoxic insecticides, such as pyrethroids, carbamates, organochlorides and organophosphates, which are quick-acting and require only 24 h to reach maximum, clothianidin needed a maximum of six days for mosquitoes to reach full susceptibility [19]. In the cone bioassays, mosquitoes exposed for longer times required fewer days to become susceptible. Test mosquitoes exposed to clothianidin for 30 min according to the WHO standard’s protocol [24] required 48 h to reach 98% mortality.

The neonicotinoids act on the nicotinic acetylcholine receptors (nAchR) of insects causing stomach poison and acute contact toxicity [19, 29], which may be responsible for mosquito delayed mortality [19]. It was observed that mosquitoes exposed to cement wall surfaces recorded slightly higher mosquito mortality than those exposed to mud wall surfaces. Previous studies [23, 30, 31] in determining factors that affect residual efficacy of insecticides revealed that, insecticides sprayed on cement wall surfaces have a high residual activity, but a short residual life as compared to mud wall surfaces, which also gives a relatively low residual activity but with a longer residual life [4, 32].

The malaria vectors from northern Ghana harboured high frequencies of *kdr* and *ace-1* mutations, suggesting resistance of mosquitoes to other classes of insecticides used in IRS. For clothianidin, delayed mortality needs to be assessed to avoid an underestimation of the efficacy and also to avoid a false reportage of vector resistance. [19, 33]. In 2019, Oxborough et al. [19] conducted a study to assess the susceptibility of malaria vectors to clothianidin in sixteen African countries, including Ghana. The findings from their study showed that, malaria vectors in Gbullung-Ghana were susceptible to clothianidin after 7 days, whereas, malaria vectors from Kumbungu-Ghana were found to be resistant. However, this current study found malaria vectors from Kumbungu to be susceptibility after 5 days. This finding corroborates with other studies [34, 35] across sub-Saharan Africa.

Since mosquitoes have become resistant to the currently used four classes of insecticides, the introduction of clothianidin for IRS in northern Ghana in 2021 offers a new perspective on controlling the resistant mosquito population. The occurrence of cross-resistance is less likely as clothianidin acts on nicotinic acetylcholine receptors, which is different from the target-sites of the four insecticide classes. Oxborough et al. [33] and Ngufor et al. [29] conducted studies to assess the susceptibility of pyrethroid-resistant *An. gambiae* to a mixture of clothianidin and deltamethrin. Their results showed that these pyrethroid-resistant mosquitoes were susceptible to clothianidin, corroborating the absence of cross-resistance. Clothianidin therefore promises to be a very effective insecticide for public health use against resistant *An. gambiae*.

The most abundant species identified was *An. gambiae* s.s, while *An. coluzzii* was modestly prevalent and *An. arabiensis* relatively scarce (< 10%). This is consistent with findings from other studies in the region [36–38]. Studies in Ghana and other parts of Africa [38–40], which ensures the larvae get adequate sunlight exposure. The higher abundance of *An. gambiae s.s*, and *An. coluzzii* was expected, given that, both species have been linked to temporary and permanent rain-dependent pools, respectively [38, 41]. The presence of *An. arabiensis* may be attributed to the dry sub-arid environment of the area. A previous study in the Northern region of Ghana noted a co-dominance *An. coluzzii* and *An. arabiensis* [38]. *Anopheles arabiensis* has a zoophilic preference, although it is also anthropophilic, which has implications for malaria vector control efforts targeting endophilic and endophagic vectors. Other integrated larval control management approaches such as biolarvicidals and environmental management may be used be to augment the impact of IRS.

## Conclusions

In this study, *An. gambiae* s.l. were susceptible to clothianidin, but this insecticide showed a delayed killing effect. The exposure duration was negatively correlated with the time required for the mosquitoes to reach maximum mortality. *Anopheles gambiae* s.l. mosquitoes harboured high frequencies of *kdr* whilst *ace-1* mutation occurred in moderation.

## Data Availability

All generated or analysed data is included in this article.

## Abbreviations

Ace: Acetylcholinesterase
DNA: Deoxyribonucleic acid
IRS: Indoor residual spraying
LLIN: Long lasting insecticidal net
NMCP: National Malaria Control Programme
PCR: Polymerase chain reaction
PMI: President’s Malaria Initiative
RH: Relative humidity
Vgsc: Voltage-gated Sodium Channels
IVCC: Innovative Vector Control Consortium
IRAC: Insecticide Resistance Action Committee
WHO: World Health Organization
